# Functional characterization of breast cancer using pathway profiles

**DOI:** 10.1186/1755-8794-7-45

**Published:** 2014-07-21

**Authors:** Feng Tian, Yajie Wang, Michael Seiler, Zhenjun Hu

**Affiliations:** 1 Center for Advanced Genomic Technology, Boston University, Boston, MA 02215, USA; 2 Core Laboratory for Clinical Medical Research, Beijing Tiantan Hospital, Capital Medical University, Beijing, P. R. China; 3 Department of Clinical Laboratory Diagnosis, Beijing Tiantan Hospital, Capital Medical University, Beijing, P. R. China

**Keywords:** Signature genes, Pathway, Pathway profile, Enrichment analysis, Breast cancer

## Abstract

**Background:**

The molecular characteristics of human diseases are often represented by a list of genes termed “signature genes”. A significant challenge facing this approach is that of reproducibility: signatures developed on a set of patients may fail to perform well on different sets of patients. As diseases are resulted from perturbed cellular functions, irrespective of the particular genes that contribute to the function, it may be more appropriate to characterize diseases based on these perturbed cellular functions.

**Methods:**

We proposed a profile-based approach to characterize a disease using a binary vector whose elements indicate whether a given function is perturbed based on the enrichment analysis of expression data between normal and tumor tissues. Using breast cancer and its four primary clinically relevant subtypes as examples, this approach is evaluated based on the reproducibility, accuracy and resolution of the resulting pathway profiles.

**Results:**

Pathway profiles for breast cancer and its subtypes are constructed based on data obtained from microarray and RNA-Seq data sets provided by The Cancer Genome Atlas (TCGA), and an additional microarray data set provided by The European Genome-phenome Archive (EGA). An average reproducibility of 68% is achieved between different data sets (TCGA microarray vs. EGA microarray data) and 67% average reproducibility is achieved between different technologies (TCGA microarray vs. TCGA RNA-Seq data). Among the enriched pathways, 74% of them are known to be associated with breast cancer or other cancers. About 40% of the identified pathways are enriched in all four subtypes, with 4, 2, 4, and 7 pathways enriched only in luminal A, luminal B, triple-negative, and HER2+ subtypes, respectively. Comparison of profiles between subtypes, as well as other diseases, shows that luminal A and luminal B subtypes are more similar to the HER2+ subtype than to the triple-negative subtype, and subtypes of breast cancer are more likely to be closer to each other than to other diseases.

**Conclusions:**

Our results demonstrate that pathway profiles can successfully characterize both common and distinct functional characteristics of four subtypes of breast cancer and other related diseases, with acceptable reproducibility, high accuracy and reasonable resolution.

## Background

Diseases are abnormal conditions of the human body resulted from significant nonlethal malfunctions that affect the human physiological system. Traditionally, diseases are characterized by pathology and observation of clinical phenotypes. Although these methods have proved successful in many applications, they lack the sensitivity to detect diseases before the appearance of symptoms and also have a limited ability to distinguish complex disease classes [1,2] which may present confusing or overlapping symptoms.

With the development of genomic technology, a promising approach to overcome limitations of the traditional method is to identify a set of genes as a genetic signature whose combined expression pattern is the uniquely characteristic of a given phenotype [3,4]. In the last decade, some gene signatures have been developed for cancers [5-9] and other diseases [10,11], indicating that the use of these signatures can assist in defining disease, predicting disease recurrence, aiding disease diagnosis and guiding treatment decision.

There are still obvious obstacles that prevent the application of gene signatures in clinical practice [12,13]. One major problem is the low reproducibility between signature genes. The overlaps of gene signatures derived from different data sets of the same disease are generally very few compared to the total number of signature genes [14], while stable gene signatures are crucial to the robustness of predictors [15]. The reasons for this discrepancy may include different cohorts of patients, different statistical methods, and different experimental technologies involved in identifying the signature genes [16]. It has been further suggested that a large number of samples are required to achieve a robust gene signature [14,15]. However, another reason for this discrepancy may lie in the observation that diseases are directly resulted from perturbed cellular functions which are generally carried out by groups of genes in the forms of complexes, modules or pathways [17]. Therefore, it is reasonable to assume that any gene whose change of expression leads to the perturbed molecular function may be a potential signature gene. This assumption is partially evidenced by the fact that both gene signatures developed in [18] and [19] can capture cell proliferation related biological processes and pathways [20], and that dysregulations of functionally related genes result in similar clinical phenotypes [21]. This assumption may also explain why sophisticated methods can rarely find much better gene signatures than simple methods [22]. From this perspective, it may be more appropriate to characterize diseases at the functional level.

Pathway-based methods have been extensively applied to analyze large-scale genome-wide data with varied purposes and applications [23-29]. Some of them classify tumor samples based on pathway-level measurements [30]; many of them, such as PWEA [31] and GSEA [32] identify perturbed pathways between two distinct phenotypes (e.g., tumor vs. normal) using expression data [28]. These enrichment methods often require a significant number of samples to achieve a statistically robust analysis. With appropriate stability and reliability, the resulting pathways of these methods may serve as reference pathways to be compared against pathways identified by sample-based analysis [33,34] in clinical applications such as disease diagnosis and personalized medicine.

In this study, we report a new approach to characterize diseases at the functional level, with our aim being to both consolidate redundant gene lists and to generate a list of pathways which is both accurate and reproducible. For a given disease, a pathway profile is generated based on the enrichment analysis of differential gene expression data between normal and tumor tissues: a binary vector whose elements indicate whether a given function (represented by a KEGG pathway) is perturbed. Using breast cancer and four clinically-relevant subdivisions (luminal A, luminal B, triple-negative and HER2+) as examples, we examine the new approach from three perspectives: to determine whether the pathway profile can be reproduced from the data generated by different technologies (Microarray vs. RNA-Seq), as well as from separate cohorts (The Cancer Genome Atlas (TCGA) vs. The European Genome-phenome Archive (EGA)), to determine whether the resulting pathways are associated with the functional perturbation resulted from the breast cancer and its subtypes, and finally to determine whether the pathway profile can distinguish different subtypes of breast cancer as well as distinguish breast cancer from other diseases. Our results indicate that the new approach achieves 68% average reproducibility between different data sets (TCGA microarray vs. EGA microarray data) and 67% average reproducibility between different technologies (TCGA microarray vs. TCGA RNA-Seq data). Among the enriched pathways, 74% of them are known to be associated with breast cancer or other cancers by extensive literature search. Approximately 40% of the pathways are enriched in all four subtypes and there are 4, 2, 4, and 7 pathways enriched only in the luminal A, luminal B, triple-negative, and HER2+ subtypes, respectively, implying that pathway profiles not only reveal shared mechanisms in the four subtypes but also outline the subtype-specific operations that may potentially be used as signature pathways to distinguish them. Comparison of profiles between subtypes, as well as other diseases including ovarian cancer, glioblastoma multiforme (GBM), and obesity, reveals that the luminal A and luminal B subtypes of breast cancer are closer to each other than to other subtypes, luminal A and luminal B subtypes are closer to the HER2+ subtype than to the triple-negative subtype, and subtypes of breast cancer are more likely to be closer to each other than to other diseases.

## Methods

### Data sources

808 tumor and 106 normal samples of TCGA RNA-Seq data (Illumina HiSeq 2000 RNA Sequencing platform) were downloaded from the TGGA portal on Oct. 2012. 522 tumor samples with available PAM50 classification [35] and 63 normal samples of TCGA microarray data (Agilent G4502A platform) were downloaded on Nov. 2012. 496 tumor and 58 normal samples overlap between the two TCGA data sets (drawn from the same patients). Both the “discovery” and “validation” EGA data sets were also downloaded, which consisted of 997 and 995 tumor samples, respectively, and 144 normal samples (Illumina HT-12 v 3 platform, accession number EGA S00000000083) [36]. The EGA discovery data set was used in our analysis and the EGA validation data set was used to further verify our major results (See discussion in Additional file 1). For brevity, the “discovery” EGA data is referred to as the EGA data set in the remainder of this paper unless otherwise stated. 37 tumor and 8 normal samples of the TCGA ovarian cancer data set (Affymetrix HG-U133A platform, batch 9) were downloaded on Feb. 2013, and 24 tumor and 10 normal samples of TCGA GBM data set (Affymetrix HG-U133A platform, batch 8) were download on Nov. 2013. The obesity data set (Affymetrix HG-U133_Plus_2 platform) of 5 obesity and 6 control samples was downloaded from GEO [GDS3688] (omental adipose from obese, prepubertal children).

No major batch effects were observed for the two TCGA breast cancer data sets [35]. The batch effect of EGA data set has been removed by a linear model [36]. To eliminate batch effects in the ovarian cancer data, only batch 9 of TCGA ovarian cancer data set was used [37]. Similarly, only batch 8 of the TCGA GBM data set was used because the possibility of batch effects within the GBM data set may not be ignored (http://bioinformatics.mdanderson.org/main/TCGABatchEffects:Overview).

A total of 269 human pathways were downloaded from KEGG [38] on Jun. 2013. 175 of these pathways in 30 pathway categories were used in our analysis after excluding all disease pathways and pathways with size either smaller than 16 or larger than 350 (to increase statistical power).

### Classification of breast cancer samples

To be consistent with clinical practice, we classified tumor samples into luminal A, luminal B, triple-negative, and HER2+ subtypes using the following steps. First, HER2+ samples were identified based on the test results of immunohistochemistry (IHC) or florescence in situ hybridization (FISH). The rest of the samples were then split into ER+ and ER- samples based on estrogen receptor (ER) status provided by IHC test results. ER+ samples were then further classified into luminal A and luminal B subtypes using PAM50 classification. Finally, triple-negative samples were extracted from the pool of ER- samples according to progesterone receptor (PR) status based on IHC test results. PAM50 classification results alone were used whenever ER or HER2 status was not available in the clinic data. As EGA data does not provide the PR status, instead we used the expression-based classification result for PR status [36]. PAM50 classification results were downloaded from the UCSC Cancer Genomics Browser [39] for TCGA data sets and the supplementary materials in its original publication [36] for the EGA data set. More details on sample classification can be found in Additional files 1 and 2.

### Pathway enrichment analysis

We focused on the enrichment of pathways abnormally perturbed in the disease state compared to the normal state in four major subtypes of breast cancer. PWEA [31] was used in this study to carry out pathway enrichment analysis, as a comprehensive study previously indicated that it has a higher sensitivity than other enrichment analysis methods including GSEA [32] with little or no loss of specificity [31,40]. The PWEA results of all data sets are provided in Additional file 3.

### Definition of reproducibility

Our measure of reproducibility was applied to both pathway and gene-based profiles. Using pathways as an example, the reproducibility *r* can be defined as

$$r=\left(\frac{N_{C}}{N}+\frac{N_{C}}{N^{'}}\right)/2$$

where *N* and *N*’ are the number of enriched pathways for two different data sets, and *N*_*c*_ is the number of overlapping of enriched pathways between two data sets.

### Pathway profile

For a given disease, a pathway profile p can be defined as:

$$p=\left[p_{1},p_{2},\ldots p_{N}\right]$$

where *N* is the total number of pathways used in the analysis and *p*_*n*_ (1 ≤ *n* ≤ *N*) is equal to either 1 or 0 to indicate whether *n*_th_ pathway is enriched or not, respectively.

### Analysis workflow

**Figure 1:**
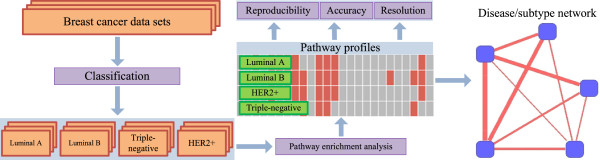
**The workflow of pathway profiling.** The tumor samples of three data sets are classified into luminal A, luminal B, triple-negative, and HER2+ subtypes by clinical data and PAM50 classification results. We then perform pathway enrichment analysis for each subtype in each data set individually using the PWEA algorithm. After that we evaluate our method based on its reproducibility, accuracy, and resolution. Finally, we build a small disease/subtype network based on the pathway profiles of each of the four subtypes of breast cancer as well as related diseases.

The sketch of our approach is depicted in Figure 1. Our approach utilized three main data sets which encompassed two separate patient cohorts and three distinct gene expression measurement platforms. The samples were first split into luminal A, luminal B, triple-negative, and HER2+ subgroups on each platform independently. Perturbed functions (represented by KEGG pathways [38]) were then identified using the PWEA algorithm [31]. Pathway profiles were constructed thereafter as binary vectors with length equal to the number of pathways with each element set to either 1 or 0 to indicate whether the corresponding pathway is enriched. Finally, correlations between breast cancer subtypes and other diseases were calculated by comparing their pathway profiles using hypergeometric statistics. The detail of our results is described below according to three key factors that may impact the performance of our analysis: reproducibility, accuracy and resolution.

## Results

Breast cancer is a complex and heterogeneous disease [41] which consists of four major clinically-relevant subtypes: luminal A, luminal B, triple-negative, and HER2+, which vary in prognosis and require distinct treatments [5,42]. Gene signatures play an important role in the classification of breast cancer subtypes. However, a comparative study of five sets of gene signatures for breast cancer indicated that the overlap between gene signatures is still low [43]. Our goal therefore is to have a stable and accurate characterization of breast cancer subtypes using pathway profiles so that the common perturbed pathways among the four subtypes, as well as specific pathways uniquely perturbed for each subtype, will be appropriately identified.

The numbers of enriched pathways for each subtype in different data sets are shown in Table 1. It is clear that, on average, the luminal A subtype has fewer enriched pathways than the other three subtypes. All of the enriched pathways are identified based on a false discovery rate (FDR) cut-off of 0.1, calculated using the Benjamini-Hochberg method [44].

**Table 1:** Number of enriched pathways for each subtype of breast cancer and data set

**Data set**	**Luminal A**	**Luminal B**	**Triple**-**negative**	**HER2+**
TCGA RNA-Seq	66	84	55	85
TCGA microarray	48	66	65	69
EGA microarray	61	87	111	103

### Reproducibility

Reproducibility is an essential requirement for almost all published scientific work. We focused on the reproducibility of our method over varying patient cohorts and platforms, with the intention to show that our result can be broadly applied in practice.

The reproducibility (See definition in Methods) of our method was evaluated using the gene expression data generated either from different expression measurement technology (Microarray vs. RNA-Seq), different sample sets (TCGA vs. EGA), or both. Only matched tumor samples (496) and normal samples (58) from each TCGA data set were used when examining the reproducibility between two technologies, and all 914 TCGA RNA-Seq samples (808 tumor and 106 normal) and 585 TCGA microarray samples (522 tumor and 63 normal) were used for the rest of the analysis.

**Figure 2:**
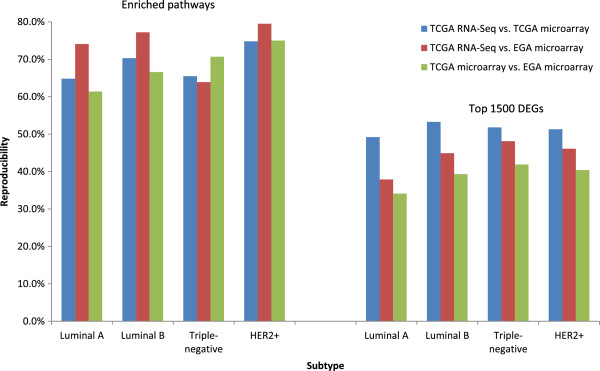
**Reproducibility of enriched pathways  (the left side) and top DEGs (the right side) between each pair of data sets for each subtype of breast cancer.** The FDR cut-off is set as 0.1 for enriched pathways. The top 1500 genes are used to calculate reproducibility for DEGs.

Overall, about 67%, 71%, 67% and 76% average reproducibility were achieved (Figure 2) for luminal A, luminal B, triple-negative, and HER2+, respectively over different data sets. This result is consistent with a previous study [45] where RNA samples were analyzed separately on 4 different microarray platforms and the percentage of overlapped functional perturbation between any two platforms fell in the range of 57-70%. On the contrary, it has been shown that any pair of gene signatures developed for breast cancer share only a few common genes [43]. For example, there are only 17 overlapping genes in the two signatures sets [14] (with 456 and 231 genes respectively) developed for breast cancer survival-related prediction [6,18]. Similarly, only three common genes have been found [46] in two sets of genetic markers (each has about 70 genes) predicting the metastasis of breast cancer [7,19] with similar accuracy. To make a more intuitive comparison with the gene-based method, we calculated the reproducibility of the top 1500 DEGs between each pair of our data sets. As shown in Figure 2, the reproducibility of each enriched pathway set is clearly much higher than that of the top DEGs for all pairs of data sets. These results further verified that pathway profiling has a much better reproducibility than gene-based methods.

#### Reproducibility across technologies

Recently, RNA-Seq has become a powerful alternative to microarrays due to advantages such as high resolution, increased dynamic range, lower background noise, relatively little technical variation, and the ability to profile the entire transcriptome [47,48]. It is therefore expected that the TCGA RNA-Seq data set may provide more reliable results than other two data sets.

We focus on the matched samples between the TCGA RNA-Seq and TCGA microarray data sets to examine the effect of gene expression measurement technology on the reproducibility of our approach. The comparison of enriched pathways generated from the two data sets results in 60%, 73%, 65% and 70% reproducibility for the luminal A, luminal B, triple-negative, and HER2+ subtypes respectively, with about 67% on average. We also found that the reproducibility based on matched samples was slightly smaller than those based on all samples except in the case of luminal B: 65%, 70%, 66%, and 75% respectively, for luminal A, luminal B, triple-negative and HER2+ subtypes as shown as blue bars in the left side of Figure 2. This observation implies that increasing the number of samples may improve reproducibility [14,15]. Meanwhile, only 45%, 53%, 50%, and 52% reproducibility was found for the top 1500 DEGs for the corresponding subtypes with 50% on average. These results indicate that the functional profile-based method presents advantages over the gene-based method and can be used to directly compare results that are generated from data sets produced by different technologies.

### Reproducibility across different data sets

We further investigate the reproducibility of our approach across different data sets. Among the three data sets used in the study, there is, as expected, the smallest difference between TCGA RNA-Seq and TCGA microarray data sets because 496 out of 522 samples from the TCGA microarray data are drawn from the same patient pool as the TCGA RNA-Seq data set, whereas the largest difference separated the TCGA RNA-Seq and EGA microarray data (different patients and different technologies). However, the corresponding average reproducibility over four subtypes is almost invariable with 69%, 68%, and 74% for the data set pairs TCGA RNA-Seq vs. TCGA microarray, TCGA microarray vs. EGA microarray, and TCGA RNA-Seq vs. EGA microarray respectively (left side of Figure 2). On the contrary, there is an obvious difference with regards to the reproducibility of the top 1500 DEGs between each dataset pair, and this is shown on the right side of Figure 2. For example, the reproducibility of top DEGs between the two TCGA data sets is always larger than the reproducibility of the other two data set pairs in all four subtypes. From this perspective, pathway profiles would appear to be better suited for comparative studies with different data sets that may be generated by different technologies.

### Determination of the appropriate data set

As mentioned at the beginning of the previous section, RNA-Seq data is expected to generate more reliable results than two other data sets [47,48]. However, in general it is challenging to determine which pathway profiles are better due to the lack of a gold standard [40,49]. Here we adopt a simple strategy to address this issue with the assumption that the pathways predicted by multiple data sets should be more reliable than those predicated by only one data set. This assumption is partly evidenced by the observation that the pathways enriched in all three data sets generally have lower *p*-value while the pathways enriched in only one data set often have higher *p*-values (more discussion can be found in Additional files 1 and 4). From these perspectives, we first generated a reference pathway profile as the benchmark where a pathway is enriched only if it is enriched in more than one data set. We then calculated the reproducibility of pathway profiles in each data set against the reference pathway profiles for each subtype. As shown in Table 2, the pathway profile resulted from the TCGA RNA-Seq data set has the highest average reproducibility. As a result of these findings, the analyses in the remainder of this paper are performed against the RNA-Seq data set unless otherwise stated.

**Table 2:** **Reproducibility between pathway profiles** (**PPs**) **of each data set and reference pathway profiles** (**RPPs**)

	**Luminal A**	**Luminal B**	**Triple-negative**	**HER2+**	**Average**
PPs of TCGA RNA-Seq data set vs. RPPs	88%	90%	79%	91%	87%
PPs of TCGA microarray data set vs. RPPs	76%	80%	89%	85%	83%
PPs of EGA microarray data set vs. RPPs	86%	86%	79%	88%	85%

### Accuracy

The accuracy of the perturbed pathways identified in our analysis is measured by their biological relevance to breast or other cancers based on survey of relevant literature sources. About 74% of the enriched pathways (enriched in at least one subtype) are known to be associated with breast cancer or cancers such as those shown in Table 3 and 4.

**Table 3:** Overlaps of common enriched pathways across three data sets

**Pathway**	**Pathway category**	**Reference**^**a**^
Glycolysis/Gluconeogenesis	Carbohydrate metabolism	[82]
Sphingolipid metabolism	Lipid metabolism	[83]
Purine metabolism	Nucleotide metabolism	[84]
Pyrimidine metabolism	Nucleotide metabolism	[85]
Arginine and proline metabolism	Amino acid metabolism	[86]
Tyrosine metabolism	Amino acid metabolism	[87]
Phenylalanine metabolism	Amino acid metabolism	[88]
One carbon pool by folate	Metabolism of cofactors and vitamins	[89]
Fanconi anemia pathway	Replication and repair	[90]
PI3K-Akt signaling pathway	Signal transduction	[91]
Regulation of actin cytoskeleton	Cell motility	[92]
Focal adhesion	Cell communication	[93]
Adipocytokine signaling pathway	Endocrine system	[94]
Progesterone-mediated oocyte maturation	Endocrine system	-
Axon guidance	Development	[95]

^a^Reference shows association between a given pathway and breast cancer or other cancers.

**Table 4:** **HER2**+ **specific pathways**

**Pathway**	**Pathway category**	**Reference**^**a**^
Tryptophan metabolism	Amino acid metabolism	-
Terpenoid backbone biosynthesis	Metabolism of terpenoids and polyketides	-
Drug metabolism - cytochrome P450	Xenobiotics biodegradation and metabolism	-
mTOR signaling pathway	Signal transduction	[63]
Serotonergic synapse	Nervous system	-
Long-term depression	Nervous system	-
Circadian rhythm	Environmental adaptation	[64]

^a^Reference shows association between a given pathway and the HER2+ subtype.

### Common enriched pathways

**Figure 3:**
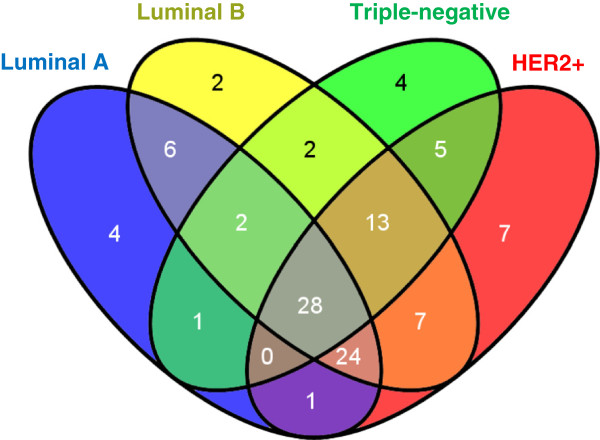
**The Venn diagram of pathway enrichment analysis results from the RNA**-**Seq data.** There are 15 regions in the Venn diagram including common enriched pathways and subtype-specific pathways. Common enriched pathways are pathways enriched in all four subtypes of breast cancer. Subtype specific pathways are pathways enriched in only one subtype of breast cancer.

As shown in Figure 3, there are 28 common enriched pathways (CEPs) across all four subtypes of breast cancer (See Additional file 5 for the whole list), which indicate the shared features of breast cancer. The CEPs occupy about 42%, 33%, 51%, and 33% of the total enriched pathways in luminal A, luminal B, triple-negative, and HER2+ subtypes, respectively. To test the reliability of CEPs, we first performed the enrichment analysis over all tumor samples without the separation of subtypes, and 86% of these CEPs are enriched in this case. We also checked enrichment analysis results in the TCGA and EGA microarray datasets, where we found that 24 of the total 28 CEPs (86%) are confirmed by at least one other data set and 15 of them are confirmed in both (Table 3). An extensive literature search indicates that 24 CEPs show relationships with either breast cancer or cancers (See Table 3 for details). These results indicate that despite the heterogeneity of breast cancer samples, different subtypes share a relatively high degree of similar molecular mechanisms to support tumor growth and metastatic dissemination. These CEPs might also be used as the “signature pathways” of breast cancer when compared to other diseases.

Among 28 CEPs, 9 are relevant to metabolism such as Glycolysis/gluconeogenesis, Purine metabolism and Tyrosine metabolism. This is not surprising, as cancer cells must perturb metabolic pathways to provide energy and building blocks to support aggressive cell growth and proliferation [50]. Thus, these pathways may account for higher biosynthesis of nucleic acids (e.g., pathways in the category of nucleotide metabolism) and proteins (e.g., pathways in the category of amino acid metabolism) and higher energy demands (e.g., pathways in the category of carbohydrate metabolism) of tumors.

We also noted that CEPs consist of some well-known cancer related pathways such as pathways in the categories of replication and repair, signal transduction, cell growth and death. For example, the CEP “Cell cycle” appears to be in agreement with the intuition that inappropriate proliferation is one of the most remarkable characteristics of the cancer cell. This is expected and can be considered as a benchmark result for our method, as several other pathway-based studies in cancer have observed similar results [51-53]. Additionally, the CEPs also contain the PI3K-Akt signaling pathway. It has been shown that many breast cancer tumors harbor mutations in the PI3K-Akt signaling pathway [35]. These mutations are thought to lead to activation of RHEB, which in turn promotes activation of the mTOR gene [54], one downstream effect of which is vastly increased protein production and much larger cancer cells. The notch signaling pathway, on the other hand, has attracted increasing attention as the potential new therapeutic targets for cancer patients [55], and our results indicate that it may also be applicable to the breast cancer.

Two hormonally-related pathways are observed to be enriched in CEPs as well, including Progesterone-mediated oocyte maturation and the Estrogen signaling pathway. As expected, we observe that Estrogen signaling is significantly upregulated in ER+ tumors (luminal A and luminal B) and significantly downregulated in triple-negative tumors. Though HER2+ tumors are not known to be directly enriched in estrogen-related genes, nevertheless we find that the pathway is still significantly dysregulated in HER2+ tumors. It has been suggested that ER+/HER2+ tumors utilize crosstalk between the MAPK signaling pathway and the ER signaling pathway to evade common anti-estrogenic therapies such as Tamoxifen [56].

#### Subtype-specific pathways

Subtype-specific pathways are especially interesting, since they are potential candidates for signature pathways for each subtype of breast cancer. As shown in Figure 3, luminal A, luminal B, triple-negative, and HER2+ subtypes have 4, 2, 4, and 7 subtype-specific pathways (about 6%, 2%, 7%, and 8% of their enriched pathways) respectively. These pathways represent the heterogeneity of breast cancer subsets.

#### Luminal A specific pathways

Luminal A has 4 subtype-specific pathways: Ubiquitin mediated proteolysis, Endocytosis, Carbohydrate digestion and absorption, and Vasopressin-regulated water reabsorption. The enrichment of Ubiquitin mediated proteolysis pathway is evidenced by the recent work [57] where it is found that the luminal A subtype had an increased expression level of cyclin D1 which regulates proteolysis mediated by ubiquitin [58].

There are two interesting pathways that are not enriched in luminal A subtype in comparison to the other three subtypes: the Homologous recombination and the p53 signaling pathways. This observation is consistent among all three data sets and may therefore be used to aid in the distinguishability of the luminal A subtype. This observation is also supported in the literature where it has been reported that a functional defect in homologous recombination is common in triple-negative breast cancer and in a subset of high grade ER and/or HER2 positive breast cancer [59]. Homologous recombination may also be associated with the luminal B subtype through the *BRCA2* gene which is known to be involved in error-free DNA repair of double-strand breaks (DSBs) through homologous recombination and BRCA2 mutation carriers have a predilection for developing breast cancers of the luminal B subtype [60]. On the other hand, the luminal A subtype has the lowest mutation frequency among the four subtypes of breast cancer [35], therefore it is not surprising that the p53 signaling pathway is not enriched in the luminal A subtype.

#### Luminal B specific pathways

The luminal B subtype has only two subtype-specific pathways: Histidine metabolism and Phosphatidylinositol signaling systems, although it has many more enriched pathways than the luminal A subtype. It should also be noted that pathways enriched by the luminal B subtype have much more overlaps with those found enriched in the triple-negative and HER2+ subtypes than in the luminal A subtype (Figure 3).

Since ER status is a very important factor in planning breast cancer treatment, we also outline the pathways that are specific to the ER+ subtype (luminal A and luminal B). Among 6 ER+ specific pathways, four of them have supporting evidence from previous studies: the Primary bile acid biosynthesis, Jak-STAT signaling pathway, Complement and coagulation cascades, and GnRH signaling pathways (More details can be found in Additional file 1).

#### Triple-negative specific pathways

The triple-negative subtype has 4 subtype-specific pathways: Alanine, aspartate and Glutamate metabolism, Lysine degradation, Vascular smooth muscle contraction, and Glutamatergic synapse. Two of these have supporting evidence in previous studies. Metabotropic glutamate receptor-1 (GRM1) has been reported as an oncogene in the progression of triple negative breast cancer [61], whose alteration may affect the pathway Alanine, aspartate and glutamate metabolism. It has been observed that the amino acid metabolism is also a major source of energy and carbon for tumor cell growth and survival in invasive breast cancer such as the triple-negative subtype [62]. Thus, the perturbation of the Lysine degradation pathway is very likely resulted from the changes in the energy metabolism of tumor cells.

#### HER2+ specific pathway

The HER2+ subtype has the largest number of subtype-specific pathways among the four subtypes as shown in Table 4, which may be due to the important role of HER2 in promoting cell growth and proliferation. Among 7 HER2+ specific pathways, mTOR signaling pathway and Circadian rhythm have supporting evidence from previous studies. For example, it was pointed out that constitutively activating HER2 and EGFR stimulated many of the same intracellular signaling proteins and pathways as wild type receptors, including the mTOR pathway [63]. Additionally, the deregulated expression of the circadian related genes PER1, PER2 and PER3 in breast cancers has been studied [64]. It was found that methylation of the PER gene promoters has a strong correlation with c-erbB2 expression.

### Resolution

The resolution of our approach aims to test whether the pathway profiles have enough detail not only to distinguish the different diseases but also to correctly assess the correlations between them based on the perturbed functions. To achieve this, we evaluate the disease correlation by calculating the hypergeometric probability of corresponding pathway profiles: a method that has been successfully applied to calculate the correlation between phylogenetic profiles in our previous study [65]. We further generated pathway profiles for ovarian cancer (TCGA, Affymetrix HG-U133A platform), GBM (TCGA, Affymetrix HG-U133A platform) and obesity (GEO, GDS3688, HG-U133_Plus_2 platform) in order to compare breast cancer against other diseases.

**Figure 4:**
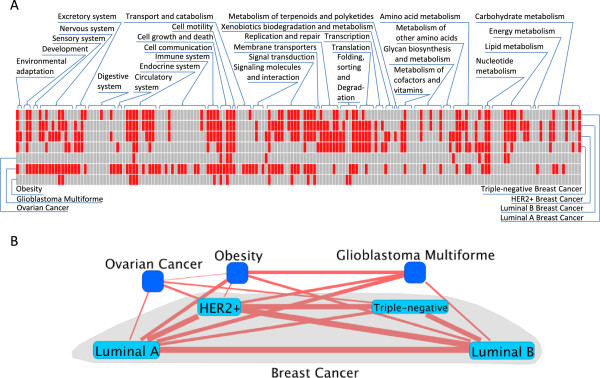
**Evaluate disease correlations by using pathway profiles. (A)** Pathway profiles for luminal A, luminal B, HER2+, and triple-negative subtypes of breast cancer, as well as ovarian cancer, glioblastoma multiforme (GBM), and obesity. Each red rectangle represents an enriched pathway. From right to left, KEGG pathways are sorted according to pathway categories and have the same order as the FDR values of enriched pathways in these 7 diseases/subtypes (Additional file 6). Due to limitation of space, here only pathway categories are labeled. This figure roughly shows that luminal A and luminal B subtypes are closer to the HER2+ subtype than the triple-negative subtype. **(B)** Disease/subtype correlation networks based on pathway profile. Each node represents one disease and subtypes are embedded in a metanode representing breast cancer [72-74]. The width of each edge is roughly proportional to the correlation between the disease/subtype pair. Here only the significant correlations (*p*-value < 0.05) are displayed (See Additional file 7). The network is drawn by the VisANT system [67-71].

The resulting profiles are drawn in Figure 4A using Gitools [66] with 11, 85, and 14 enriched pathways for ovarian cancer, GBM, and obesity respectively according to PWEA results (See Additional file 6). Figure 4B shows the correlations between seven diseases/subtypes as a small network drawn by VisANT [67-71] where 4 subtypes are encapsulated by the metanode [72-74] of breast cancer and the edge thickness is roughly proportional to correlation strength. With a 0.05 *p*-value cutoff, a total of 17 disease pairs exhibit significant correlation with varied strength (See Additional file 7). As expected, most of the correlations between four subtypes are stronger than those between subtypes and the other three diseases. Among the four subtypes, luminal A and luminal B subtypes are closer to each other than to the other two subtypes. On the other hand, the luminal A and luminal B subtypes are closer to the HER2+ subtype than to the triple-negative subtype, which may not be surprising because the HER2+ subtype is simply characterized by copy number variation in the HER2 amplicon, while it has been suspected that ER+ (luminal A and luminal B) and ER- subtypes may have differing cells of origin [75]. Similarly, both triple-negative and HER2+ subtypes have stronger correlation to ovarian cancer than luminal A and luminal B subtypes have, most likely because of the effect of P53 gene [35]. Meanwhile, the fact that luminal B, triple-negative, and HER2+ subtypes are more aggressive tumors than the luminal A subtype is reflected by the observation that both triple-negative and HER2+ subtypes are closer to the luminal B subtype than the luminal A subtype. We also find the luminal A subtype is closer to obesity than to ovarian cancer, indicating the potential connection between breast cancer and obesity through estrogen [76]. Interestingly, our results show that GBM is significantly correlated with luminal A, luminal B, HER2+ breast cancer and obesity. It was pointed out that sex hormones are important in the growth of breast cancer and are also important in the development of GBM [77]. Furthermore, the anti-estrogen drug Tamoxifen has been found to be effective in decreasing glioblastoma cell proliferation [78]. On the other hand HER2 is the most frequently expressed tyrosine kinase receptors in GBM cells [79]. The association between GBM and obesity may be explained by the important role of leptin in both of these two diseases. The current model suggests that obesity in human is due to a desensitization to leptin while within gliomas, there is a correlation between tumor grade and tumor expression of leptin and its receptor [80].

## Discussion

**Figure 5:**
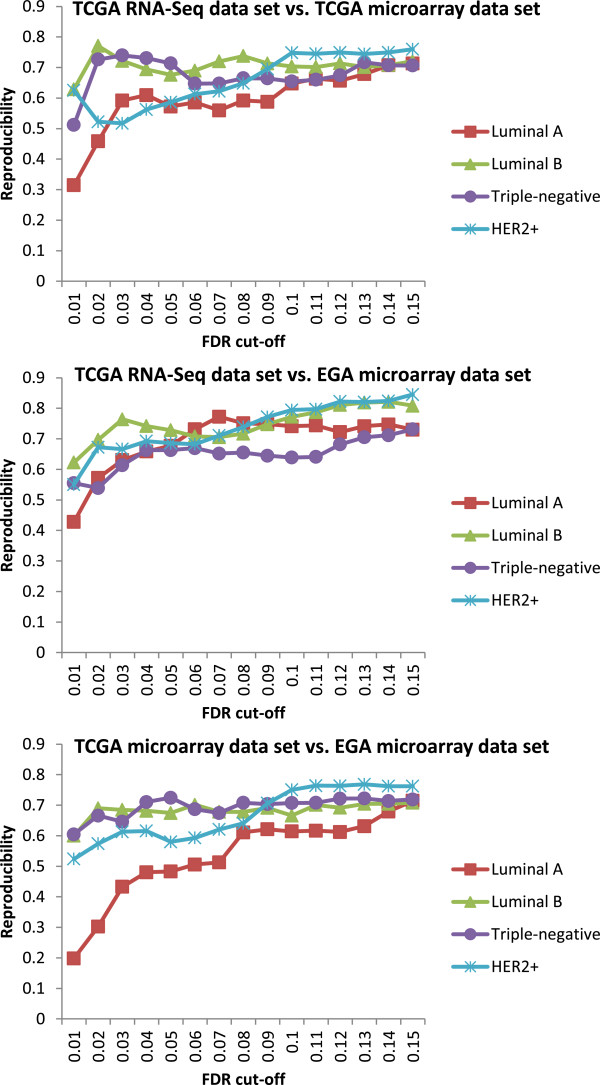
**Reproducibility of enriched pathways between different data sets/platforms vs. FDR cut-off for each subtype of breast cancer.** In general, the reproducibility increases quickly at the beginning and gradually becomes flat as FDR cut-off increases.

All the enriched pathways in this study are identified based on a FDR cut-off 0.1. This cut-off was optimized to achieve reasonable reproducibility (Figure 5) while maintaining adequate coverage and accuracy. More detail on the FDR cut-off is addressed in Additional file 1. Another important factor that needed to be taken into account is the coverage of KEGG pathway genes with the corresponding gene expression measurement technology. All three data sets used in this study have a good coverage of KEGG pathway genes (TCGA RNA-Seq, TCGA microarray and EGA microarray data sets contain about 20360, 17814 and 17621 genes, corresponding to the 97%, 91% and 90% coverage of 5584 total KEGG pathways genes, respectively), which however may not hold for the large sets of microarray array data available in GEO databases. More discussion of the pathway coverage can also be found in Additional file 1.

The comparison of the reproducibility resulted from the pathway profiles and top DEGs are in general conservative. From a hypergeometric statistics perspective, a larger number of genes resulted from the different analyses will achieve better reproducibility. We therefore used the top 1500 DEGs in the reproducibility comparison instead of known signature genes available in the literature because the number of the latter is generally much smaller than 1500. In addition, we also performed the comparison using the top 6000 DEGs and the enriched pathways still achieves better reproducibility (See details in Additional file 1). The robustness of our approach is further verified by using the EGA validation data set [36]. There is little change in the reproducibility between two TCGA data sets and the EGA data set when substituting the EGA discovery set with the validation set (in Additional file 1 Figure S5 where more discussion of the EGA validation set can also be found).

Despite the improvement over classical gene-based methods in characterizing breast cancer and related diseases, this approach has great potential to be further enhanced. First, it depends on the prior knowledge of pathways which are far from complete, and identifying new pathways is a difficult, time-consuming, and labor intensive work. An alternative approach to partially ameliorate this limit is to replace pathways with functional modules that may be computationally identified [81]. Second, current pathway profiles are represented as a simply binary vector that may be improved by incorporating some additional information such as the corresponding statistical significance of the enriched pathway to make each profile more quantitative. For a simple approach, one may directly set elements of pathway profile as the *p*-values or enrichment scores of given pathways.

## Conclusion

Reproducibility is one of the main challenges for the identification of gene signatures. Besides technical factors, the disparity may result from the fact that diseases are directly caused by the perturbations of the molecular function that are generally carried out by a set of genes in the form of modules or pathways. Therefore it may be more appropriate to characterize diseases at the functional level than at the gene level. Following this perspective, we developed a novel approach to characterize diseases using the pathway profiles and evaluated the approach based on profiles’ reproducibility, accuracy and resolution. Using four subtypes of breast cancer as an example, the results of this new approach are promising with 70% average reproducibility, 74% average accuracy (e.g., references in Table 3[82-95]) and reasonable resolution to identify the correlations between not only different diseases, but also their subtypes.

## Abbreviations

TCGA: The Cancer Genome Atlas; EGA: The European Genome-phenome Archive; GBM: Glioblastoma multiforme; DEGs: Differentially expressed genes; FDR: False discovery rate; PPs: Pathway profiles; RPPs: Reference pathway profiles; CEP: Common enriched pathway.

## Competing interests

The authors declare that they have no competing interests.

## Author’s contributions

FT and YW designed the method and experiments. FT carried out the computation and both FT and YW performed the analysis. FT drafted the manuscript. MS normalized TCGA breast cancer RNA-Seq data, provided constructive advice, and revised the manuscript. ZH supervised the project, gave suggestions, and revised the manuscript. All authors read and approved the final manuscript.

## Pre-publication history

The pre-publication history for this paper can be accessed here:

http://www.biomedcentral.com/1755-8794/7/45/prepub

## Supplementary Material

Additional file 1 Supplementary materials on determination of ER, PR, and HER2 status, differentially expressed genes, determination of FDR cut-off, coverage of KEGG pathway genes, comparison between consistent and inconsistent pathways, EGA validation data set, and ER+ specific pathways. Click here for file

Additional file 2 Lists of samples for three data sets and four subtypes of breast cancer. Click here for file

Additional file 3 PWEA results of all used data sets. Click here for file

Additional file 4 Lists of consistent and inconsistent pathways across three data sets for four subtypes of breast cancer. Click here for file

Additional file 5 Lists of CEPs and subtype specific pathways. Click here for file

Additional file 6 **FDR of enriched pathways for pathway profiles shown in Figure **4**A.** Click here for file

Additional file 7 ***p*-values for subtype/disease correlations shown in Figure **4**B.** Click here for file
