# Supplementary material for: Functional characterization of breast cancer using pathway profiles
Source: BMC Med Genomics. 2014 Jul 21;7:45. doi: 10.1186/1755-8794-7-45 (PMC4113668; doi:10.1186/1755-8794-7-45)
Supplement: Additional file 1 — Supplementary materials on determination of ER, PR, and HER2 status, differentially expressed genes, determination of FDR cut-off, coverage of KEGG pathway genes, comparison between consistent and inconsistent pathways, EGA validation data set, and ER+ specific pathways. [file 1755-8794-7-45-S1.pdf]

Supplementary materials

Determination of ER status by clinic data. The clinical data for TCGA data sets is downloaded from the UCSC Cancer Genomics Browser [1] and the TCGA original publication for breast cancer data [2]. If the ER status of a sample is not available, we determine its ER status according to PAM50 classification results downloaded from the UCSC Cancer Genomics Browser. In this case, we consider a sample as ER+, if it belongs to luminal subtypes.

The clinical data and PAM50 classification results for EGA data set are provided by the original publication for EGA data [3]. If the ER status of a sample is not available, we determine its ER status according to PAM50 classification results as introduced above.

Determination of PR status. PR status is used to select triple-negative samples from ER- samples. For TCGA data sets, we just ignore a sample, if its PR status is not provided by clinical data. The original publication for EGA data [3] does not provide the PR status based on immunohistochemistry (IHC) test. The PR status of EGA samples is determined by an expression-based classification method *MCLUST* [4], whose classification results are provided in the original publication for EGA data [3].

Determination of HER2 status. To improve the clinical calls of HER2 status for TCGA data, the HER2_Final_Status is derived (Supplemental Table 1 in [2]) by using both clinical data and DNA copy number data. For the TCGA samples contained by the Supplemental Table 1 in [2], we firstly determine their HER2 status according to the field HER2_Final_Status. For those samples contained by the Supplemental Table 1 in [2] whose HER2 status cannot be determined by the field HER2_Final_Status, we determine their HER2 status according to PAM50 classification results. We consider a sample as HER2+, if it belongs to the HER2-enriched subtype. For those samples which are not contained by the Supplemental Table 1 in [2], we determine their HER2 status by the following steps.

Step 1. If the IHC value is 0 or 1+, the HER2 status of the given sample is negative. If the IHC value is 3+, the HER2 status of the given sample is positive. If the HER2 status of the given sample cannot be determined by IHC value, go to Step 2.

Step 2. Determine the HER2 status according to the results of florescence in situ hybridization (FISH). If the HER2 status of the given sample cannot be determined by FISH results, go to Step 3.

Step 3. Determine the HER2 status according to the filed IHC_status in the TCGA clinical data. If the HER2 status of the given sample cannot be determined by IHC_status, go to Step 4.

Step 4. Determine the HER2 status according to PAM50 classification results. We consider a sample as HER2+, if it belongs to the HER2-enriched subtype.

During above steps, we firstly use IHC values and FISH results to determine HER2 status instead of directly using IHC_status in clinical data, because it has been pointed that the clinical call (IHC_status) is not always consistent with IHC values and FISH results according to current clinical guidelines for determining HER2 status of breast cancer [2].

For EGA data set, if HER2 status cannot be determined by clinical data (field HER2_IHC_status in the table S2 and S3 in [3]), PAM50 classification results are used as introduced above.

Annotate EGA data sets. It has been noted that many Illumina probes have unreliable original annotations, which leads to sub-optimal performance for the downstream biology analysis [6]. As a consequence, we re-annotated probes of EGA data according to the results in [6] and only used the probes with either 'Perfect' or 'Good' annotation. When multiple probes target the same gene, we used the median of these probes.

Differentially expressed genes. We used two-tailed *t*-test to identify differentially expressed genes (DEGs) for each subtype of breast cancer in each data set. After *p*-values for all genes were computed, we computed the FDR to correct for multiple testing problem using the method by Benjamini and Hochberg [7].

The comparison of the reproducibility between enriched pathways and top 1500 DEGs is based on following concerns. 1) From a hypergeometric statistics perspective, larger number of genes will achieve better reproducibility. We therefore used the top 1500 DEGs in the reproducibility comparison instead of known signature genes available in the literature because the number of the latter is much smaller than 1500. In addition, the selection of 1500 is based on our observation that the increase in reproducibility of the top DEGs slows down quickly after its number reaches 1500 (Figure S1). 2) We also performed the comparison using the top 6000 DEGs and the enriched pathways still achieve better reproducibility (Figure S2), while the total KEGG pathways used in this study only covers 5584 genes.

Determination of FDR cut-off. In this study, we used FDR cut-off 0.1 to select enriched pathways. In general, decreasing the cut-off value will increase the percentage of true positives, but also decrease common enriched pathways (CEPs) across different data sets and increase false negatives. Empirically, how to determine cut-off depends on the application. For our case, we intended to characterize subtypes of breast cancer by all the dysregulated pathways instead of finding the most significant biomarkers. Thus we considered the following points when determining FDR cut-off. First, results of each data set should contain a reasonable number of enriched pathways for further analysis. Using a too stringent DFR cut-off may miss some potentially significant results. Second, results of different data sets should have a nontrivial overlap. Third, by literature search we found that quite a few of enriched pathway are related to breast cancer or cancer when using this cut-off value. In some cases, the pathway enrichment analysis result may have no significant pathways or too many significant pathways when using a general FDR cut-off value. Then one may use the top ranked pathways for analysis. This strategy can still make sense in some extent. In addition, some applications require the same number of enriched pathways for different data sets. In this case, people can determine the appropriate number of top pathways by making sure that the top pathways of each data set can all satisfy the same constraint of FDR cut-off.

Coverage of KEGG pathway genes. In this work, all three used breast cancer data sets have a good coverage of pathway genes. For pathway enrichment analysis, a fundamental requirement is that most of the pathway genes should be contained by the platform. The used KEGG pathways contain 5584 genes. TCGA RNA-Seq, TCGA microarray and EGA microarray data sets contain about 20360, 17814 and 17621 genes, which cover 97%, 91% and 90% pathways genes, respectively. We also investigated how many genes of an individual pathway were covered by platforms. We defined the pathway gene

coverage rate for a pathway as the percentage of genes of a pathway which are contained by a data set. We then plotted the curves of the empirical cumulative distribution function (CDF) for the pathway gene coverage rate for different data sets as shown in Figure S3. It is clearly seen that all three data sets have a good coverage of genes for each pathway. For TCGA RNA-Seq, TCGA microarray and EGA microarray data sets, only 0.6%, 5.7% and 9.7% pathways have pathway gene coverage rates smaller than or equal to 80%, respectively. Currently, there is still no a standard to judge whether a platform is acceptable when considering coverage of pathway genes. An experiential method is to compare the CDF for pathway gene coverage rate of a platform with that of a widely used platform such as the Affymetrix HG-U133A chip. The platform will be acceptable, if its CDF curve for pathway gene coverage rate is about in the right of that of the Affymetrix HG-U133A chip. In Figure S3 we can see that all the three used breast cancer data sets have better pathway gene coverage than that of the Affymetrix HG-U133A chip.

Comparison between consistent and inconsistent pathways. We have grouped enriched pathways according to the number of data sets in which they are enriched in Additional file 4 so that it will be much easier to identify the enriched pathways that are consistent or inconsistent across the three data sets. After carefully observing these consistent or inconsistent results, we think that the consistent pathways (enriched in all data sets) are more likely to be true positive results than those inconsistent ones in general because 1) we find that consistent pathways generally have lower *p*-values and the pathways enriched in only one data set generally have higher *p*-values (Figure S4); 2) it is much harder to find literature support for pathways enriched in only one data set than others.

EGA validation data set. The EGA data [3] has an additional validation data set besides the discovery data set used in our analysis. We have generated pathway profiles using EGA validation data set. We found that the reproducibility of the enriched pathways between EGA discovery and validation data sets are 81%, 87%, 91% and 89% for luminal A, luminal B, triple-negative, and HER2+ subtypes, respectively. As expected, the reproducibility between EGA discovery set and validation set is higher than the rest cases reported in the manuscript of all the four subtypes of breast cancer (Figure S5). As also shown in Figure S5, there is little change in reproducibility between the two TCGA data sets and the EGA data set when substituting the EGA discovery set with the validation set. The another reason why the EGA discovery set is used in the manuscript is because the discovery set seems more homogenerous than the validation set. For an example, we found 17% tumor samples in EGA validation data set are in the normal-like subtype based on PAM50 classification. This percentage is much higher than that of other three data sets, which are 3%, 2%, and 6% for TCGA RNA-Seq data set, TCGA microarray data set, and EGA discovery data set, respectively. Currently, there are still doubts about the real existence of normal-like subtype and some researchers believe they could be a technical artifact from high contamination with normal tissue [8]. Taken these factors together, the EGA discovery data set is therefore used in the manuscript.

ER+ specific pathways. ER+ specific pathways are those pathways specific to both of luminal A and luminal B subtypes, which are all ER+ tumors. It is valuable to have an insight on these ER+ specific pathways, as ER status is a very important factor in planning breast cancer treatment. Among 6 ER+ specific pathways listed in Table S1, four of them have supporting evidence from previous studies: the

Primary bile acid biosynthesis, Jak-STAT signaling pathway, Complement and coagulation cascades, and GnRH signaling pathway. Lower levels of unconjugated bile acids have been observed in the serum of women with ER+ tumors than that in the healthy women [9]. Further study [10] suggested that naturally occurring bile acids influence the growth and steroid receptor function of human breast cancer cells based on the experiments in the ER+ MCF-7 human breast cancer cell line. For the Jak-STAT signaling pathway, the presence of pStat5 is found predominantly in well-differentiated estrogen receptor (ER) –positive tumors and is associated with favorable prognosis [11]. The complement and coagulation cascades pathway has also been shown to be involved specifically with ER+ breast cancer, where one study [12] noted that this pathway was enriched for proteins in plasma samples of ER+ breast cancer compared to control plasma, where 467 quantified proteins were mapped to their corresponding genes to do pathway enrichment analysis. For the GnRH signaling pathway, it has been demonstrated that estradiol positive and negative feedback on GnRH neuron firing activity require signaling via ERα [13], which is overexpressed in ER+ tumors.

References

1. Cline MS, Craft B, Swatloski T, Goldman M, Ma S, Haussler D, Zhu J: **Exploring TCGA Pan-Cancer data at the UCSC Cancer Genomics Browser**. *Sci Rep* 2013, **3**:2652.
2. Koboldt DC, Fulton RS, McLellan MD, Schmidt H, Kalicki-Veizer J, McMichael JF, Fulton LL, Dooling DJ, Ding L, Mardis ER *et al*: **Comprehensive molecular portraits of human breast tumours**. *Nature* 2012, **490**(7418):61-70.
3. Curtis C, Shah SP, Chin SF, Turashvili G, Rueda OM, Dunning MJ, Speed D, Lynch AG, Samarajiwa S, Yuan YY *et al*: **The genomic and transcriptomic architecture of 2,000 breast tumours reveals novel subgroups**. *Nature* 2012, **486**(7403):346-352.
4. Fraley C, Raftery AE: **Model-based clustering, discriminant analysis, and density estimation**. *Journal of the American Statistical Association* 2002, **97**(458):611-631.
5. Ein-Dor L, Zuk O, Domany E: **Thousands of samples are needed to generate a robust gene list for predicting outcome in cancer**. *Proceedings of the National Academy of Sciences of the United States of America* 2006, **103**(15):5923-5928.
6. Barbosa-Morais NL, Dunning MJ, Samarajiwa SA, Darot JF, Ritchie ME, Lynch AG, Tavare S: **A re-annotation pipeline for Illumina BeadArrays: improving the interpretation of gene expression data**. *Nucleic Acids Res* 2010, **38**(3):e17.
7. Benjamini Y, Hochberg Y: **Controlling the False Discovery Rate - a Practical and Powerful Approach to Multiple Testing**. *Journal of the Royal Statistical Society Series B-Methodological* 1995, **57**(1):289-300.
8. Eroles P, Bosch A, Perez-Fidalgo JA, Lluch A: **Molecular biology in breast cancer: intrinsic subtypes and signaling pathways**. *Cancer Treat Rev* 2012, **38**(6):698-707.
9. Baker PR, Reid AD, Smith GJ, Yong S, Stenzel DJ, Preece PE, Wood RAB, Cuschieri A: **Bile-Acids and Estrogen-Receptor Activity in Breast-Cancer**. *Biochemical Society Transactions* 1987, **15**(6):1056-1057.
10. Baker PR, Wilton JC, Jones CE, Stenzel DJ, Watson N, Smith GJ: **Bile-Acids Influence the Growth, Estrogen-Receptor and Estrogen-Regulated Proteins of Mcf-7 Human Breast-Cancer Cells**. *British Journal of Cancer* 1992, **65**(4):566-572.
11. Sansone P, Bromberg J: **Targeting the interleukin-6/Jak/stat pathway in human malignancies**. *Journal of clinical oncology : official journal of the American Society of Clinical Oncology* 2012, **30**(9):1005-1014.

12. Amon LM, Pitteri SJ, Li CI, McIntosh M, Ladd JJ, Disis M, Porter P, Wong CH, Zhang Q, Lampe P *et al*: **Concordant Release of Glycolysis Proteins into the Plasma Preceding a Diagnosis of ER+ Breast Cancer**. *Cancer Research* 2012, **72**(8):1935-1942.
13. Christian CA, Glidewell-Kenney C, Jameson JL, Moenter SM: **Classical estrogen receptor alpha signaling mediates negative and positive feedback on gonadotropin-releasing hormone neuron firing**. *Endocrinology* 2008, **149**(11):5328-5334.

Figures

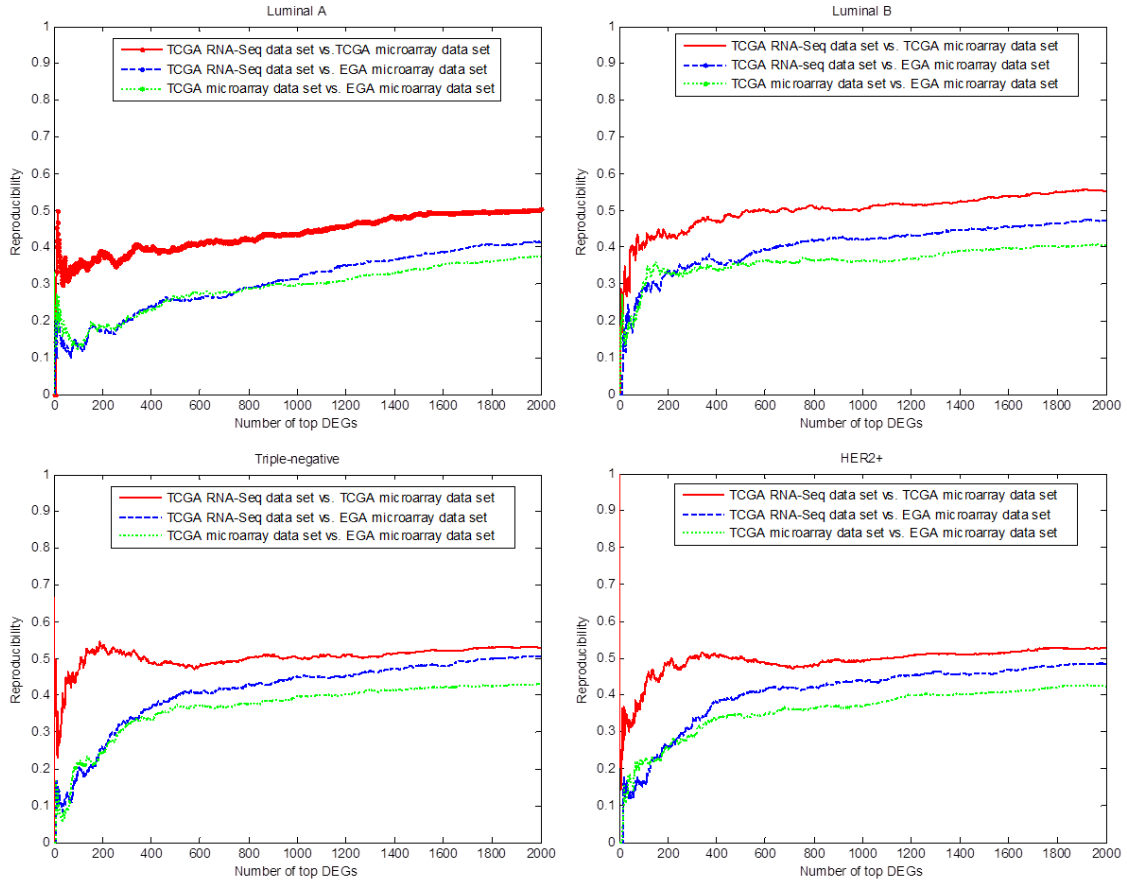

Figure S1 Reproducibility of top DEGs between different data sets vs. number of top DEGs for each subtype of breast cancer. The reproducibility of top DEGs is plotted against the number of top DEGs. In general, the reproducibility increases as the number of DEGs increases. On the other hand, the reproducibility increases more slightly even though the number of top DEGs still keeps increasing. It is clear that for each subtype of breast cancer the pair between two TCGA data sets has the highest reproducibility and the pair between TCGA microarray and EGA microarray data sets has the lowest reproducibility when the number of top DEGs is larger than some value.

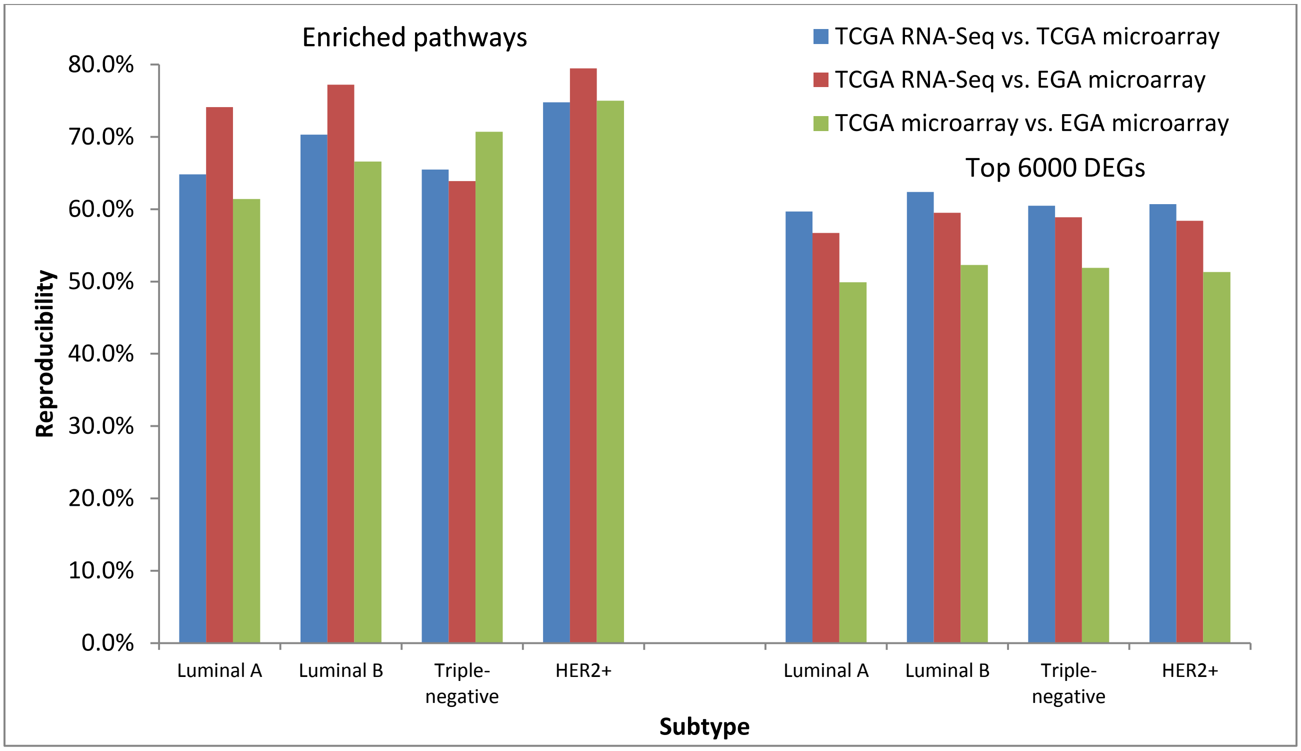

Figure S2 Reproducibility of enriched pathways (the left side) and top DEGs (the right side) between each pair of data sets for each subtype of breast cancer. The FDR cut-off is set as 0.1 for enriched pathways. Top 6000 genes are used to calculate reproducibility for DEGs.

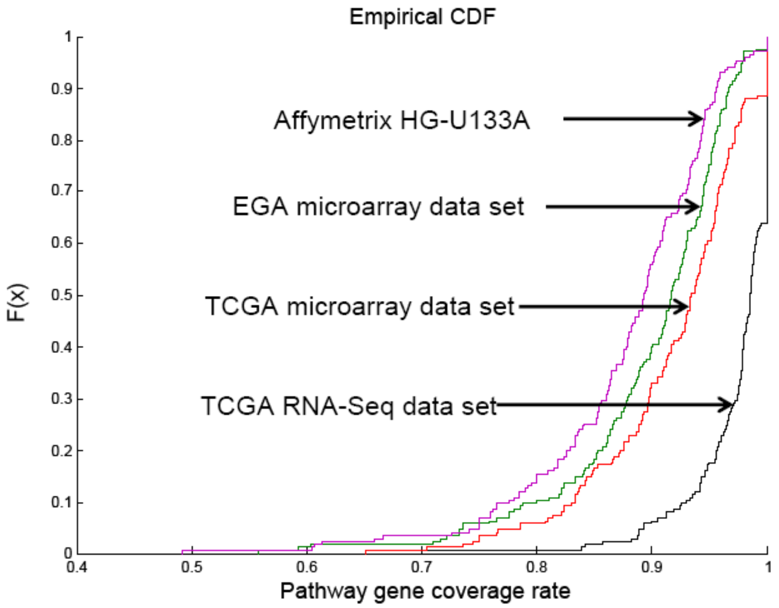

Figure S3 Curves of the empirical cumulative distribution function (CDF) for the percentage of genes of a pathway which are contained by different data sets/platforms. The pathway gene coverage rate is defined as the percentage of genes of a pathway which are covered by a data set/platform. X-axis is the pathway gene coverage rate. Y-axis represents the percentage of used KEGG pathways whose pathway gene coverage rates are smaller or equal to the corresponding pathway gene coverage rate.

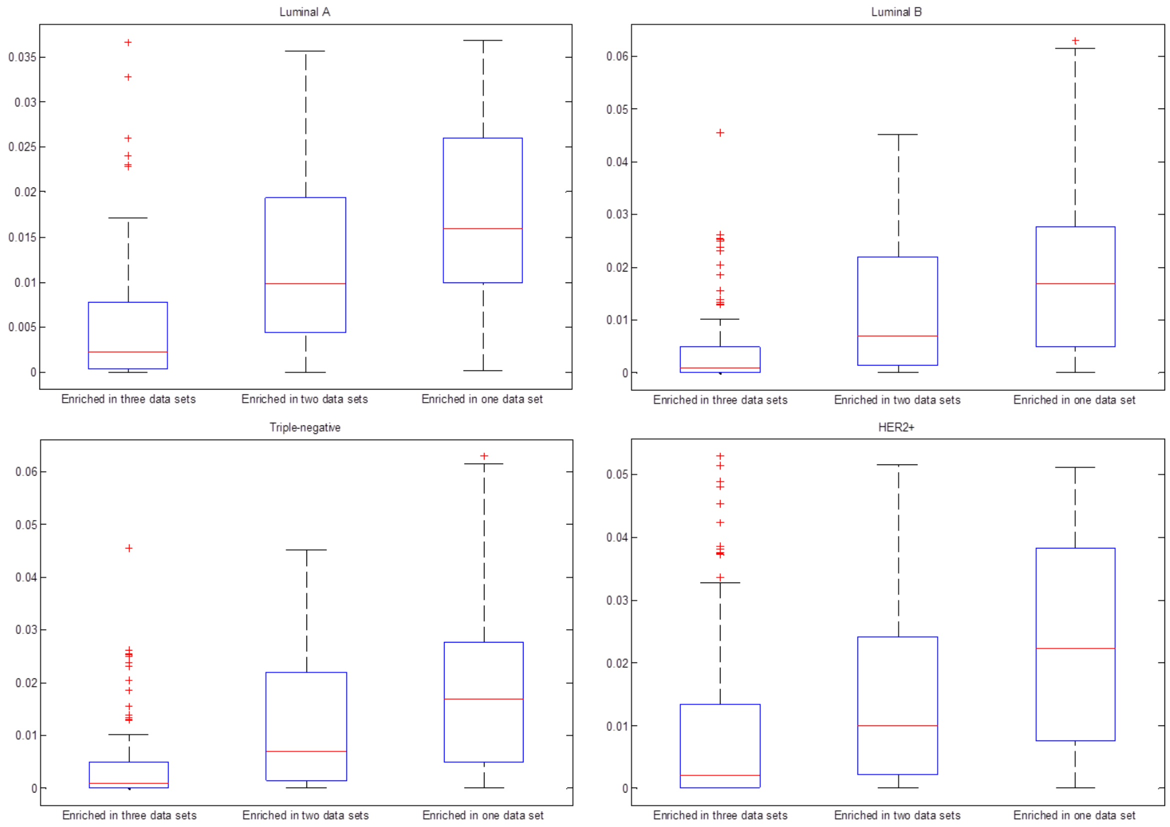

Figure S4 Boxplot of *p*-values of enriched pathways for four subtypes of breast cancer. Enriched in three, two, and one data sets represent those pathways which are enriched in three, two, and only one data sets. Note that for those pathways not enriched in all three data sets, we only count their *p*-values in the data sets where they are enriched. We can observe that in general pathways enriched in three data sets have lowest *p*-values and pathways enriched in only one data set have the highest *p*-values.

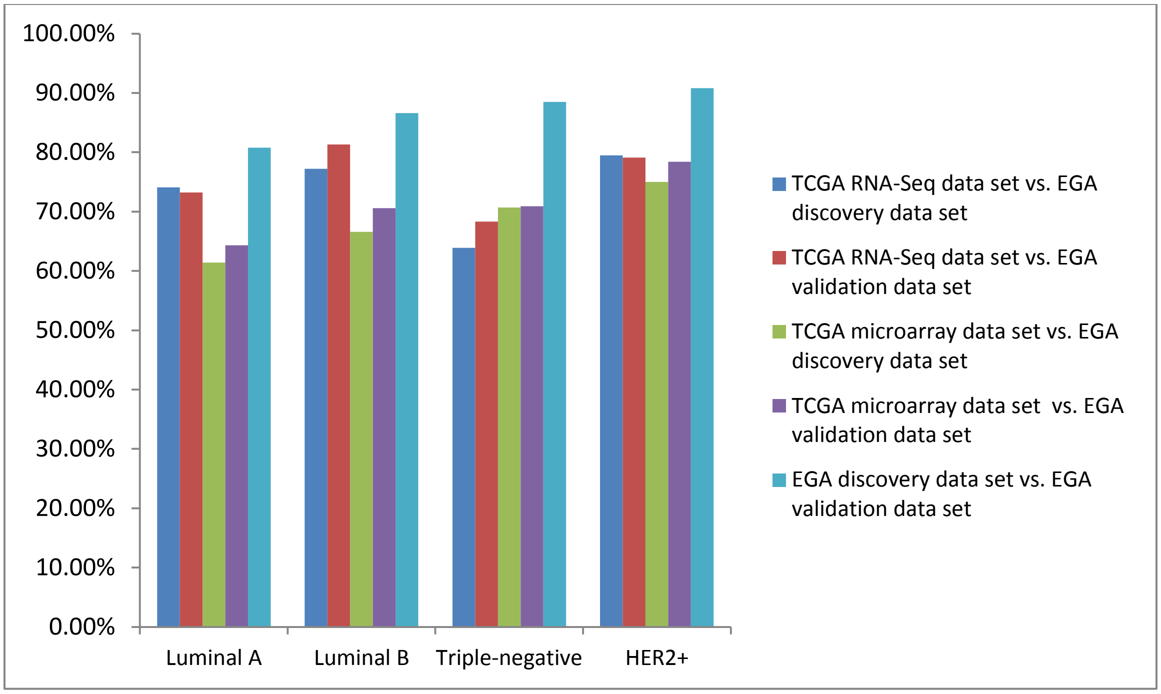

Figure S5 Reproducibility between two TCGA data sets (RNA-Seq and microarray) and two EGA microarray data sets (discovery set and validation set). The FDR cut-off 0.1 is used. It can be seen that whether using the EGA discovery set or validation set does not affect the reproducibility between TCGA data sets and EGA data sets much. On the other hand, the reproducibility between two EGA data sets is obviously higher than that between TCGA data sets and EGA data sets.

Tables

Table S1 ER+ specific pathways

**Pathway**	**Category**	**Reference[a]**
Primary bile acid biosynthesis	Lipid Metabolism	[9, 10]
Glycosaminoglycan biosynthesis - chondroitin sulfate	Glycan Biosynthesis and Metabolism	-
Jak-STAT signaling pathway	Signal transduction	[11]
Complement and coagulation cascades	Immune System	[12]
GnRH signaling pathway	Endocrine system	-
Cholinergic synapse	Nervous system	-

[a]Reference shows association between a given pathway and ER+ subtypes (luminal A and luminal B).
